# Retinal image quality with multifocal, EDoF, and accommodative intraocular lenses as studied by pyramidal aberrometry

**DOI:** 10.1186/s40662-021-00258-y

**Published:** 2021-10-06

**Authors:** Jorge L. Alio, Francesco D’Oria, Francesca Toto, Joan Balgos, Antonio Palazon, Francesco Versaci, Jorge L. Alio del Barrio

**Affiliations:** 1 Vissum Innovation, Alicante, Spain; 2 Division of Ophthalmology, Universidad Miguel Hernández, Vissum Miranza, c/ Cabañal, 1, 03016 Alicante, Spain; 3 Section of Ophthalmology, Department of Basic Medical Science, Neuroscience and Sense Organs, University of Bari, Bari, Italy; 4 Department of Clinical Medicine, Miguel Hernández University, San Juan de Alicante, Spain; 5 R&D Department, Costruzione Strumenti Oftalmici (CSO), Florence, Italy

**Keywords:** Cataract surgery, Pyramidal aberrometry, Multifocal IOL, Extended depth of focus, Accommodative IOL

## Abstract

**Background:**

To study and compare the clinical optical image quality following implantation with different premium IOLs by analysing the point spread function (PSF) Strehl ratio using a pyramidal wavefront sensor (PWS)-based aberrometer.

**Methods:**

This study included 194 eyes implanted with: (a) 19 AcrySof SA60AT (control group); (b) 19 Miniwell; (c) 24 LENTIS Mplus LS-313 MF30; d) 33 LENTIS Mplus LS-313 MF15; (e) 17 AkkoLens Lumina; (f) 31 AT LISA Tri 839MP; (g) 20 Precizon Presbyopic; (h) 20 AcrySof IQ PanOptix; (i) 11 Tecnis Eyhance. Main outcome measures were PSF Strehl ratio, PSF Strehl ratio excluding second-order aberrations (PSFw2), total root mean square (RMS), low-order aberration (LOA) and high-order aberration (HOA) RMS measured by PWS aberrometer.

**Results:**

AT LISA Tri had the highest PSFw2 Strehl ratio at both 3.0- and 4.0-mm pupil size (0.52 ± 0.14 and 0.31 ± 0.10; *P* < 0.05), followed by SA60AT (0.41 ± 0.11 and 0.28 ± 0.07) and PanOptix (0.4 ± 0.07 and 0.26 ± 0.04). AT LISA Tri was found to provide a significantly better retinal image quality than PanOptix at both 3.0 mm (*P* < 0.0001) and 4.0 mm (*P* = 0.004). Mplus MF15 was found to be significantly better than Mplus MF30 at both 3.0 mm (*P* < 0.0001) and 4.0 mm (*P* = 0.002). Total RMS, LOA RMS, HOA RMS, PSF Strehl ratio and PSFw2 varied significantly between the studied groups (*P* < 0.001).

**Conclusions:**

Far distance clinical image quality parameters measured by PWS aberrometer differed significantly according to the technology of the implanted lens. AT LISA Tri, SA60AT and PanOptix showed the highest values of far distance retinal image quality, while the lowest PSFw2 Strehl ratios were displayed by Miniwell, Mplus MF30 and Precizon Presbyopic.

## Background

Cataract surgery can be considered not only as a therapeutic but also as a refractive procedure, by the replacement of the natural opacified crystalline lens by an intraocular lens (IOL). On the other hand, an eventual degradation of the retinal image after cataract surgery, excluding corneal irregularity or loss of transparency, could be attributed not only to the IOL optical properties, but also to an imbalance of the compensatory effect between the positive spherical aberration of the cornea and the negative spherical aberration of the crystalline lens, which might be relevant when young patients are operated [1, 2].

The "ex vivo" optical bench through-focus image quality analysis and the clinical visual performance in real patients by study of the defocus curves after different types of IOL implantation has been investigated by Plaza-Puche et al., and it was found to be significantly correlated [3]. This correlation makes it necessary to study the quality of IOL both in vitro and in vivo for different models, and at different pupillary diameters [3, 4].

The selection of the IOL according to its optical and aberrometric characteristics, especially when considering the use of multifocal or extended depth of focus (EDoF) implants, is a relevant concept. Any IOL should ideally minimize ocular wavefront aberrations and optimize the retinal image quality [5], to prevent the impact that a low retinal image can have in visual quality and contrast sensitivity: this aspect can influence patient’s satisfaction after cataract surgery and interfere with the entire neuroadaptation process [6].

Wavefront aberrometry describes the individual optical characteristics of low- and high-order aberrations (LOA and HOA, respectively) in Zernike polynomials. The point spread function (PSF) and the Strehl ratio provide information on the overall optical performance of the human eye: PSF is the irradiance distribution of light from a point source projected onto the retina and it indicates the extent of blurring of the retinal image; the Strehl ratio is the ratio of the peak height of the PSF and to the maximum attainable intensity using an ideal diffraction limited optical system [7].

However, the interpretation of the aberrometric metrics in pseudophakic patients has been different in many cases and different models of aberrometers, with different working principles such as the most commonly used Hartmann–Shack method, Tscherning principle, or Ray-tracing, which have been used to measure wavefront aberration profiles; [7–11] however, these aberrometers present several limitations after multifocal IOL implant [12]. A new pyramidal wavefront sensor (PWS) has been developed and validated and it helps improve the aberration compensation efficiency by sensing aberrations [13–15].

The big advantage of a PWS is that the wavefront is sampled at the very last measuring stage, and this allows for a much higher resolution. For instance, a Hartmann–Shack sensor discretizes the wavefront at the lenslet stage and the number of lens on the lenslet determines the number of measured samples: for Hartmann–Shack, we usually have 1000–2000 lenses with a resolution of 250–1250 µm. Osiris, on the contrary has a resolution of 41 µm (45,000 samples at maximum pupil size). Consequently, Osiris can measure sharp edges (like the ones on a diffractive lens) and evaluate their effect on the PSF. These optical advantages have relevant clinical implications in the performance of this new type of aberrometry on clinical basis in refractive and lens surgery.

The present study aimed to evaluate the clinical optical quality of the eye with different types of IOLs in vivo: monofocal spherical, multifocal, EDoF or accommodative IOLs implantation, by studying the PSF Strehl ratio in the far distance image with a model of PWS-based aberrometer.

## Methods

### Study design

This is a prospective, consecutive, comparative, case series study. Inclusion criteria were uncomplicated cataract or refractive lens exchange surgery and the absence of any comorbidity or anatomical feature such as keratoconus and corneal scars that could limit the visual potential and the light transmission in the study eye. Exclusion criteria included amblyopia, axial length over 25.0 mm, any type of corneal opacity, previous ocular surgery including corneal or refractive surgery, chronic or recurrent uveitis, acute ocular disease or external/internal infection, diabetes mellitus with retinal changes. Cases with confirmed or suspicion of optic nerve damage due to glaucoma were excluded. Eyes should have postoperative corrected distance visual acuity (CDVA) of 0.1 logMAR or better and excellent foveal fixation, normal pseudophakia, total absence of posterior capsule opacification or have already performed Nd:YAG capsulotomy prior to the instrumental optical quality evaluation. Only eyes with mean preoperative keratometry between 42.5 and 45 D, spherical aberration within ± 0.25 μm, corneal HOA < 0.5 µm and angle kappa < 0.4 mm were included in the study. Cases with intraocular complications that could affect IOL performance or IOL stability were excluded, as well as any capsular changes that would increase the risk of decentration or tilt of the IOL, such as zonular weakness or pseudoexfoliation syndrome. All procedures adhered to the tenets of Helsinki Declaration of the World Medical Association. Institutional research ethical board commettee approval was obtained from our institution for the purpose of this clinical investigation.

### Clinical evaluation and optical quality

Assessment after 3 months using the same photopic conditions for all the studied IOLs included: uncorrected distance visual acuity (UDVA) and CDVA in logMAR, uncorrected near visual acuity (UNVA) and corrected near visual acuity (CNVA) at 40 cm in logMAR, and manifest refraction. In case of neuroadaptation failure, defined as a subjective reduction in this quality of vision, generally owing to perception of blurred vision, dysphotopsia or photic phenomena, explantation was decided when symptoms were unacceptable and were creating disability for the normal performance of the patient’s life. Explantation was decided between the third and sixth postoperative month. In such cases, the clinical and optical quality evaluation of those patients was assessed immediately prior to the IOL exchange. No corneal refractive surgical procedures of any type were performed either during the cataract surgery or along the follow-up of the patients included in this investigation until the end of the study protocol, at the end of the third month.

All patients had an evaluation of the optical quality of the far distance image using the PWS-based aberrometer Osiris (Costruzione Strumenti Oftalmici [CSO], Firenze, Italy). To perform measurements with Osiris, pupils were dilated using 0.5% tropicamide. In all cases, three consecutive acquisitions were obtained and the following aberrometric parameters were analysed: total root mean square (RMS), LOA RMS, HOA RMS and PSF Strehl ratio. For the purpose of the measurement of the far distance retinal image quality based on PSF Strehl ratio, the second-order aberrations were eventually excluded in the analysis of the PSF Strehl ratio (PSFw2) to avoid any bias induced by residual ametropia and delete its effect on the PSF as it is not related itself with the optical performance of the IOL, and the calculation of this parameter was based only in the magnitude of HOA present in each eye. For a given pupil radius, the second-order aberration removal is performed by decomposing the wavefront in terms of Zernike. The wavefront can thus be expressed as:

$$WF\left(\rho ,\theta \right)=\left(\sum {c}_{n}^{m}{Z}_{n}^{m}\left(\rho ,\theta \right)\right)+R\left(\rho ,\theta \right)$$

where ${Z}_{n}^{m}\left(\rho ,\theta \right)$ is the Zernike polynomial with order (n, m), ${c}_{n}^{m}$ is the coefficient resulting in the fitting and $R\left(\rho ,\theta \right)$ is the residual. The removal of the second-order can simply be performed by setting ${c}_{2}^{-2}= {c}_{2}^{0}= {c}_{2}^{+2}=0$ or in the previous formula or by subtracting ${c}_{2}^{-2}{Z}_{2}^{-2}\left(\rho ,\theta \right)+{c}_{2}^{0}{Z}_{2}^{0}\left(\rho ,\theta \right)+{c}_{2}^{+2}{Z}_{2}^{+2}\left(\rho ,\theta \right)$ from the overall wavefront.

All aberrometric parameters mentioned above were calculated for the far distance image for two different pupil diameters of 3.0 and 4.0 mm.

### The osiris pyramidal aberrometry system

The Osiris aberrometer bases its working principle on a high-resolution four-faced PWS. By comparing the light intensity among four images of the entrance pupil of the patient (known as sub-pupils), we are able to obtain measurements of the wavefront and refractive error. The wavefront error as well as the refractive error is sampled with 45,000 points at maximum pupil, which corresponds to a resolution of 41 µm. The device uses an extended light-emitting diode source emitting at 850 nm as a measurement on visible light would have dramatically reduced the pupil size. The results are then converted to the 585 nm wavelength correcting the effect of longitudinal chromatic aberration. The software shows the direct outcome of the device and due to its dense resolution, does not need Zernike polynomial based modal reconstruction to retrieve the ocular wavefront shape.

A PWS-based aberrometer measures the slopes of a wavefront focusing the foveal source image on the top of a pyramidal prism with a large apex angle. The prism acts to split the beam into 4 parts creating “sub-pupils”. The “sub-pupils” will be identical in case of aberration free wavefront and in the presence of optical aberrations, the intensity distribution among the pupils will be a function of the first derivative of the wavefront along x- and y-axis. It has been demonstrated that for each point, the derivative along the x-axis is proportional to the difference between the left and right sub-pupils, and the one along the y-axis is proportional to the difference between the top and bottom sub-pupils. The wavefront error matrix is finally calculated with a numerical integration method starting from the directional derivatives. Nevertheless, a Zernike fitting is available during the analysis process for back-compatibility with previous devices and to split the overall wavefront error in Zernike main components [13]. The Osiris aberrometer provides accurate and repeatable measures of LOAs and HOAs, even in the challenging cases of peripheral retina and multifocal optics [15].

### IOLs studied

All patients underwent microincisional cataract surgery as described in a previous study by our group [4]. Each patient received one out of nine different IOL implants (Table 1): monofocal spherical AcrySof SA60AT as control group (Alcon, Inc., group A); EDoF Miniwell (SIFI, group B); multifocal refractive LENTIS Mplus LS-313 MF30 (Oculentis GmbH, group C); multifocal refractive LENTIS Mplus LS-313 MF15 (Oculentis GmbH, group D); AkkoLens Lumina accommodative intraocular lens (AkkoLens Clinical B.V., Breda, The Netherlands, group E); multifocal diffractive AT LISA Tri 839 MP (Carl Zeiss Meditec, group F); multifocal refractive Precizon Presbyopic (Ophtec BV, group G); multifocal diffractive AcrySof IQ PanOptix Trifocal (Alcon, Inc., group H); new aspheric monofocal Tecnic Eyhance (Johnson & Johnson Vision, Inc., group I). For all participants, the IOL types implanted were selected based on the patient’s lifestyle and surgeon’s advice as well as patient’s need and preferences to decide if they may benefit more from near vision than from good intermediate vision. The IOL power selected was targeted to emmetropia. The Lumina AkkoLens accommodative IOL was implanted in the context of an independent clinical trial (P16-006-V1) [16, 17].

**Table 1:** Summary of studied groups

Group	IOL	n (RLE/CS)
Group A	AcrySof SA60AT	19 (10/9)
Group B	SIFI Miniwell	19 (13/6)
Group C	LENTIS Mplus LS-313 MF30	24 (17/7)
Group D	LENTIS Mplus LS-313 MF15	33 (19/14)
Group E	AkkoLens Lumina	17 (10/7)
Group F	AT LISA Tri 839 MP	31 (24/7)
Group G	Precizon Presbyopic	20 (12/8)
Group H	AcrySof IQ PanOptix	20 (11/9)
Group I	Tecnis Eyhance	11 (4/7)

*RLE* refractive lens exchange; *CS* cataract surgery

### Statistical analysis

Absolute and relative frequencies were calculated to describe the qualitative variables, whereas means and standard deviations were used for quantitative ones. For non-time-dependent variables, the Chi-squared test (Pearson or Fisher) and ANOVA were estimated to determine differences between groups. However, for time-dependent variables, paired t-tests and mixed linear models were used. The type I error was fixed at 5%. To detect a significant difference in the postoperative values of PSFw2 (the main outcome) with 80%, 90% and 95% power, 11, 14 and 16 eyes per group would have been necessary, respectively [18]. All calculations were made using IBM SPSS Statistics v. 25 and R v. 3.5.1.

## Results

### Demographics characteristics and clinical data

A total of 194 eyes of 120 cataract patients aged between 40 and 84 years (63.5 ± 9.7 years) were included in this study. They were grouped according to the type of pseudophakic IOL implanted, in nine different groups (Table 1). There were no intraoperative complications such as posterior capsule rupture or any postoperative complications such as IOL tilt or decentration in any of these patients included in the study. Table 2 showed the patient's demographics characteristics and clinical variables of the analysed groups. These groups were well matched at baseline in terms of sex and IOL power (*P* > 0.05), but not for age (*P* = 0.013) and laterality (*P* < 0.001). Miniwell (group B) had the highest rate of IOL substitution due to patient dissatisfaction related to neuroadaptation failure (6 eyes of 3 patients, 31.6%, *P* = 0.002).

**Table 2:** Non-time-dependent clinical variables of the analysed intervention groups

Variable	Group A n (%)/x ± s	Group B n (%)/x ± s	Group C^*^ n (%)/x ± s	Group D^*^ n (%)/x ± s	Group E n (%)/x ± s	Group F n (%)/x ± s	Group G n (%)/x ± s	Group H n (%)/x ± s	Group I n (%)/x ± s	*P*-value
Gender female	9 (75.0)	5 (45.5)	9 (42.9)	14 (53.8)	3 (33.3)	12 (75.0)	6 (60.0)	6 (60.0)	2 (33.3)	0.334
Age (years)	70.4 ± 7.1	64.6 ± 11.0	61.9 ± 7.8	60.9 ± 9.2	62.7 ± 13.8	63.9 ± 6.2	61.4 ± 11.9	60.0 ± 8.2	74.8 ± 9.9	0.013
Laterality:										
Only left eye	2 (16.7)	0 (0)	7 (33.3)	11 (42.3)	1 (11.1)	1 (6.3)	0 (0)	0 (0)	1 (16.7)	< 0.001
Only right eye	3 (25.0)	3 (27.3)	11 (52.4)	8 (30.8)	0 (0)	0 (0)	0 (0)	0 (0)	0 (0)	
Both	7 (58.3)	8 (72.7)	3 (14.3)	7 (26.9)	8 (88.9)	15 (93.8)	10 (100)	10 (100)	5 (83.3)	
IOL power (D)	20.4 ± 4.3	21.1 ± 2.9	22.1 ± 3.8	22.5 ± 3.8	20.4 ± 5.9	22 ± 3.2	21.4 ± 2.7	20.8 ± 3.7	20.8 ± 2.9	0.426
Nd:YAG laser	4 (21.1)	2 (10.5)	1 (4.2)	1 (3)	3 (17.6)	2 (6.5)	0 (0)	0 (0)	0 (0)	0.068
IOL substitution	0 (0)	6 (31.6)	1 (4.2)	4 (12.1)	1 (5.9)	0 (0)	0 (0)	2 (10)	0 (0)	0.002

*IOL* intraocular lens; *Nd:YAG* neodymium-doped yttrium aluminium garnet; *n(%)* absolute frequency (relative frequency); *x ± s* mean ± standard deviation

Group A: AcrySof SA60AT; Group B: SIFI Miniwell; Group C: LENTIS Mplus LS-313 MF30; Group D: LENTIS Mplus LS-313 MF15; Group E: AkkoLens Lumina; Group F: AT LISA Tri 839MP; Group G: Precizon Presbyopic; Group H: AcrySof IQ PanOptix; Group I: Tecnis Eyhance

Frequencies, means, and standard deviations were calculated for the total cases who received the intervention in the indicated eye in the specific group

*There was one patient whose left eye was included in Group C and the right eye in Group D

Table 3 showed the visual acuity and manifest refraction at both near and far distances. All treated groups had an improvement in UDVA, but it was not statistically significant in AkkoLens (group E, *P* = 0.068). Regarding CDVA, even if all treated groups reached a mean visual acuity between 0.86–1.03 logMAR, a postoperative improvement was only observed in SA60AT (group A, *P* < 0.001), Mplus MF15 (group D, *P* = 0.016) and PanOptix (group H, *P* = 0.019); nevertheless, the wide range of preoperative CDVA makes statistical implication about postoperative improvement less meaningful, as both patients undergoing cataract surgery and refractive lens exchange surgery have been included. All groups with the exception of group A (SA60AT) and group D (Mplus MF15) had an improvement in UNVA: postoperative data of UNVA of group D might result from the hyperopic refractive error and higher standard deviation of the spherical component in that group.

**Table 3:** Time-dependent variables of the analysed intervention groups

Variable	Group A *P*-value x ± s	Group B *P*-value x ± s	Group C^*^ P-value x ± s	Group D^*^ P-value x ± s	Group E *P*-value x ± s	Group F *P*-value x ± s	Group G *P*-value x ± s	Group H *P*-value x ± s	Group I *P*-value x ± s	*P*-value (groups)
UDVA (logMAR)	< 0.001	0.002	< 0.001	< 0.001	0.068	< 0.001	< 0.001	< 0.001	0.019	0.204
Preintervention	0.58 ± 0.24	0.38 ± 0.36	0.48 ± 0.27	0.5 ± 0.28	0.38 ± 0.38	0.42 ± 0.32	0.44 ± 0.33	0.5 ± 0.31	0.36 ± 0.38	0.726
Postintervention	0.12 ± 0.27	0.16 ± 0.22	0.09 ± 0.20	0.10 ± 0.24	0.20 ± 0.30	0.03 ± 0.12	0.08 ± 0.16	0.08 ± 0.19	0.14 ± 0.27	< 0.001
CDVA (logMAR)	< 0.001	0.392	0.082	0.016	0.059	0.087	0.053	0.019	0.111	0.003
Preintervention	0.20 ± 0.31	0.08 ± 0.26	0.07 ± 0.25	0.09 ± 0.22	0.08 ± 0.40	0.03 ± 0.28	0.08 ± 0.21	0.10 ± 0.28	0.11 ± 0.24	0.043
Postintervention	0.03 ± 0.23	0.05 ± 0.18	0.02 ± 0.12	0.04 ± 0.20	-0.01 ± 0.15	-0.01 ± 0.08	0.01 ± 0.13	0.02 ± 0.15	0.07 ± 0.21	0.033
Sphere (D)	0.553	0.753	0.261	0.343	0.938	0.020	0.858	0.102	0.125	0.036
Preintervention	− 0.75 ± 4.61	− 0.01 ± 2.69	0.97 ± 3.80	1.12 ± 3.39	− 0.62 ± 6.02	1.21 ± 2.52	0.55 ± 2.13	− 1.33 ± 4.11	− 0.82 ± 1.69	0.140
Postintervention	− 0.16 ± 0.43	0.18 ± 0.52	0.06 ± 0.48	0.50 ± 1.67	− 0.50 ± 0.83	0.14 ± 0.29	0.63 ± 0.46	0.28 ± 0.45	− 0.05 ± 0.57	0.001
Cylinder (D)	0.004	0.736	0.747	0.093	0.457	< 0.001	0.153	0.002	0.602	0.073
Preintervention	− 0.95 ± 0.57	− 0.75 ± 0.69	− 0.64 ± 0.57	− 0.93 ± 1.23	− 0.74 ± 0.82	− 0.77 ± 0.54	− 0.64 ± 0.71	− 0.61 ± 0.38	− 0.89 ± 0.81	0.758
Postintervention	− 0.42 ± 0.38	− 0.70 ± 0.85	− 0.58 ± 0.55	− 0.61 ± 0.63	− 0.91 ± 0.66	− 0.28 ± 0.45	− 0.40 ± 0.45	− 0.24 ± 0.32	− 0.75 ± 0.72	0.003
UNVA (logMAR)	0.380	< 0.001	0.023	0.086	< 0.001	< 0.001	< 0.001	< 0.001	0.002	< 0.001
Preintervention	0.61 ± 0.32	0.83 ± 0.24	0.34 ± 0.31	0.39 ± 0.29	0.66 ± 0.28	0.48 ± 0.26	0.92 ± 0.20	1.08 ± 0.13	0.59 ± 0.26	< 0.001
Postintervention	0.89 ± 0.21	0.39 ± 0.27	0.19 ± 0.18	0.30 ± 0.22	0.23 ± 0.20	0.15 ± 0.18	0.23 ± 0.17	0.21 ± 0.20	0.18 ± 0.26	< 0.001
Add (D)	0.518	< 0.001	< 0.001	0.001	0.279	< 0.001	0.058	< 0.001	0.004	< 0.001
Preintervention	2.75 ± 0.15	2.34 ± 0.40	2.37 ± 0.81	2.23 ± 1.41	2.19 ± 0.84	2.45 ± 0.49	2.18 ± 0.84	2.40 ± 0.46	1.82 ± 1.26	0.239
Postintervention	2.58 ± 0.78	1.54 ± 0.74	0.73 ± 0.96	0.90 ± 0.84	1.93 ± 0.26	0.12 ± 0.36	1.28 ± 2.00	0.25 ± 0.62	1.14 ± 1.05	< 0.001
CNVA (logMAR)	0.050	0.349	0.509	0.019	0.027	0.837	0.190	0.363	0.015	0.015
Preintervention	0.17 ± 0.29	0.13 ± 0.28	0.09 ± 0.26	0.11 ± 0.22	0.11 ± 0.25	0.11 ± 0.16	0.17 ± 0.22	0.17 ± 0.27	0.21 ± 0.13	0.205
Postintervention	0.06 ± 0.14	0.09 ± 0.19	0.11 ± 0.14	0.17 ± 0.19	0.03 ± 0.12	0.11 ± 0.08	0.12 ± 0.14	0.13 ± 0.20	0.12 ± 0.20	< 0.001

*Add* near addition; *CDVA* corrected distance visual acuity; *CNVA* corrected near visual acuity; *D* diopters; *logMAR* logarithm of the minimum angle of resolution; *UDVA* uncorrected distance visual acuity; *UNVA* uncorrected near visual acuity; *x* ± *s* mean ± standard deviation

Group A: AcrySof SA60AT; Group B: SIFI Miniwell; Group C: LENTIS Mplus LS-313 MF30; Group D: LENTIS Mplus LS-313 MF15; Group E: AkkoLens Lumina; Group F: AT LISA Tri 839MP; Group G: Precizon Presbyopic; Group H: AcrySof IQ PanOptix; Group I: Tecnis Eyhance

*There was one patient whose left eye was included in Group C and the right eye in Group D

AT LISA Tri (group E, *P* = 0.027) and Eyhance (group I, *P* = 0.015) had a significant improvement in CNVA; Mplus MF15 had a significant reduction in CNVA (group D, *P* = 0.019).

### Postoperative aberrations

**Fig. 1:**
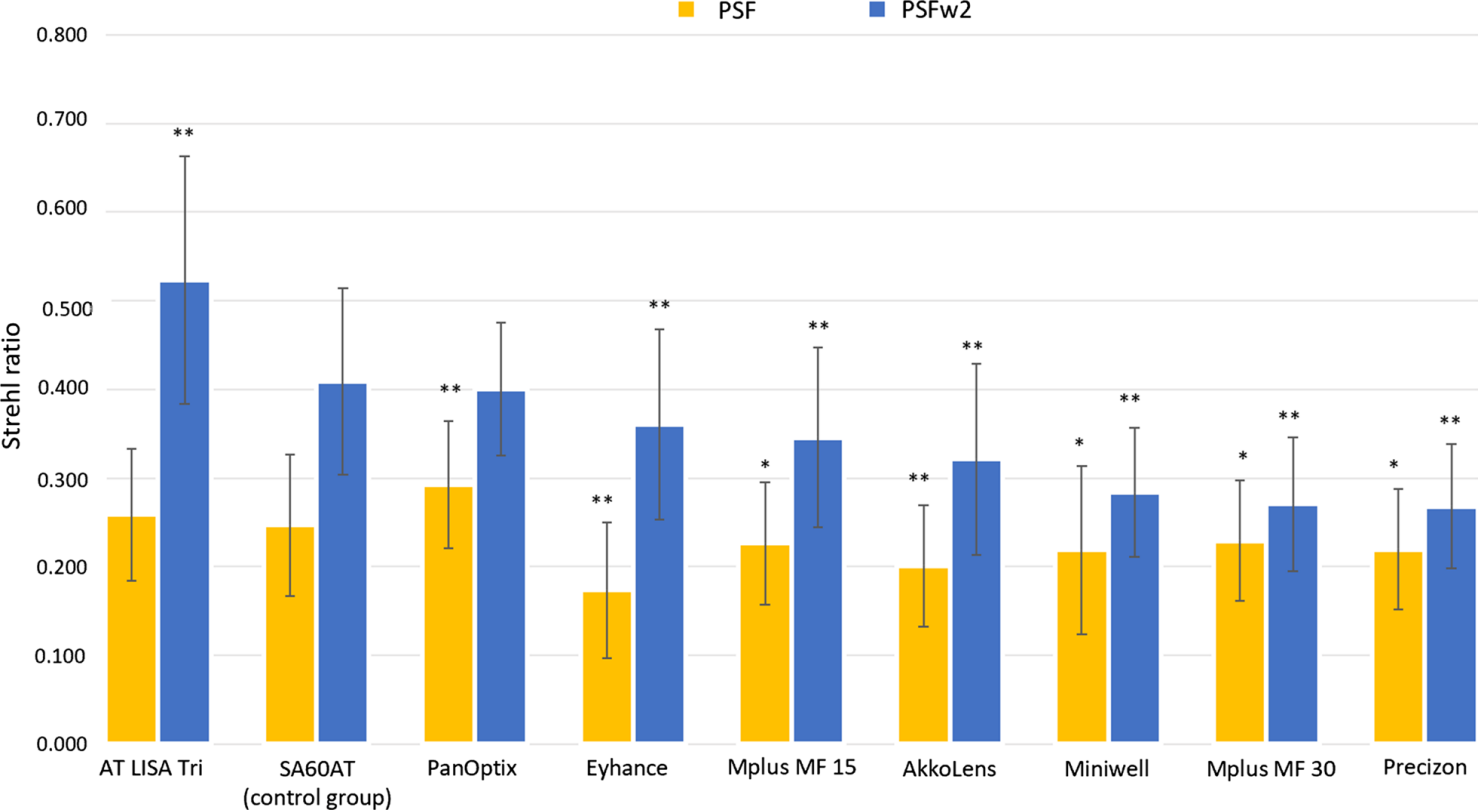
PSF Strehl ratio with and without low-order aberration for each group, obtained with a pyramidal wavefront sensor-based aberrometer, and level of significance compared to the monofocal spherical control group. *P < 0.05*,* **P < 0.001

Wavefront aberrations in the far distance image were compared for each of the nine lens types at two different pupil sizes (3.0 and 4.0 mm). Total RMS, LOA RMS, HOA RMS, PSF Strehl ratio, PSFw2 Strehl ratio were recorded and analysed. Overall, the grouping outcomes of postoperative aberrations of the far distance image with 3.0 mm and 4.0 mm pupil diameters are presented in Table 4. In terms of postoperative aberrations in the study groups, a between-group ANOVA reveal a statistically significant difference for all the values at both 3.0 and 4.0 mm pupil diameters (all *P* < 0.001). In addition, all the studied aberrations varied significantly as pupil diameter increase (*P* < 0.05). When looking to the PSFw2 Strehl ratio, AT LISA Tri (group F) had the highest significant PSFw2 Strehl ratio at both 3.0 and 4.0 mm pupil sizes (0.52 ± 0.14 and 0.31 ± 0.1), followed by SA60AT (group A, 0.41 ± 0.11 and 0.28 ± 0.07) and PanOptix (group H, 0.4 ± 0.07 and 0.26 ± 0.04). Figure 1 shows the values of PSF Strehl ratio with and without LOA, obtained at 3.0 mm, and the significance compared to the monofocal control group A. AT LISA Tri (group F) had a significant higher value of PSFw2 than the monofocal control group A (*P* < 0.0001), PanOptix (group H) was not significantly different (*P* = 0.345), while all the other groups have a significant lower PSFw2 than group A (*P* < 0.0001).

**Table 4:** Postoperative values of wavefront aberrations of the analysed intervention groups at two pupil sizes obtained at 3 months

Variable	Group A *P*-value x ± s	Group B *P*-value x ± s	Group C^*^ P-value x ± s	Group D^*^ P-value x ± s	Group E *P*-value x ± s	Group F *P*-value x ± s	Group G *P*-value x ± s	Group H *P*-value x ± s	Group I *P*-value x ± s	*P*-value (groups)^†^
Total RMS (µm)	< 0.001	< 0.001	< 0.001	< 0.001	< 0.001	< 0.001	< 0.001	< 0.001	< 0.001	< 0.001
3 mm	0.43 ± 0.19	0.44 ± 0.19	0.49 ± 0.18	0.46 ± 0.15	0.49 ± 0.16	0.33 ± 0.11	0.53 ± 0.17	0.28 ± 0.10	0.60 ± 0.28	< 0.001
4 mm	0.66 ± 0.17	0.70 ± 0.31	0.83 ± 0.21	0.83 ± 0.28	1.00 ± 0.24	0.59 ± 0.25	0.78 ± 0.22	0.42 ± 0.17	1.10 ± 0.51	< 0.001
LOA RMS (µm)	< 0.001	< 0.001	< 0.001	< 0.001	< 0.001	< 0.001	< 0.001	< 0.001	< 0.001	< 0.001
3 mm	0.39 ± 0.20	0.34 ± 0.20	0.38 ± 0.16	0.39 ± 0.16	0.42 ± 0.17	0.30 ± 0.12	0.47 ± 0.16	0.23 ± 0.11	0.56 ± 0.27	< 0.001
4 mm	0.62 ± 0.20	0.59 ± 0.26	0.64 ± 0.25	0.71 ± 0.32	0.90 ± 0.28	0.55 ± 0.25	0.64 ± 0.21	0.33 ± 0.19	1.05 ± 0.52	< 0.001
HOA RMS (µm)	< 0.001	< 0.001	< 0.001	< 0.001	< 0.001	< 0.001	< 0.001	< 0.001	< 0.001	< 0.001
3 mm	0.16 ± 0.05	0.23 ± 0.10	0.30 ± 0.13	0.21 ± 0.07	0.22 ± 0.09	0.12 ± 0.05	0.25 ± 0.08	0.15 ± 0.04	0.18 ± 0.06	< 0.001
4 mm	0.26 ± 0.08	0.38 ± 0.18	0.50 ± 0.12	0.40 ± 0.13	0.40 ± 0.14	0.20 ± 0.09	0.44 ± 0.13	0.23 ± 0.05	0.33 ± 0.14	< 0.001
PSF Strehl ratio	< 0.001	< 0.001	< 0.001	< 0.001	< 0.001	< 0.001	< 0.001	< 0.001	< 0.001	< 0.001
3 mm	0.25 ± 0.08	0.22 ± 0.09	0.23 ± 0.07	0.23 ± 0.07	0.20 ± 0.07	0.26 ± 0.07	0.22 ± 0.07	0.29 ± 0.07	0.17 ± 0.08	< 0.001
4 mm	0.18 ± 0.05	0.16 ± 0.05	0.16 ± 0.05	0.15 ± 0.04	0.12 ± 0.04	0.18 ± 0.05	0.15 ± 0.04	0.21 ± 0.05	0.12 ± 0.05	< 0.001
PSFw2 Strehl ratio	< 0.001	< 0.001	< 0.001	< 0.001	0.015	< 0.001	< 0.001	< 0.001	< 0.001	< 0.001
3 mm	0.41 ± 0.11	0.28 ± 0.07	0.27 ± 0.08	0.35 ± 0.10	0.32 ± 0.11	0.52 ± 0.14	0.27 ± 0.07	0.40 ± 0.07	0.36 ± 0.11	< 0.001
4 mm	0.28 ± 0.07	0.21 ± 0.06	0.18 ± 0.06	0.21 ± 0.06	0.23 ± 0.16	0.31 ± 0.10	0.17 ± 0.04	0.26 ± 0.04	0.21 ± 0.06	< 0.001

*HOA* high-order aberration; *LOA* low-order aberration; *PSF* point spread function; *PSFw2* point spread function without second-order aberrations; *RMS* root mean square; *x ± s* mean ± standard deviation

Group A: AcrySof SA60AT; Group B: SIFI Miniwell; Group C: LENTIS Mplus LS-313 MF30; Group D: LENTIS Mplus LS-313 MF15; Group E: AkkoLens Lumina; Group F: AT LISA Tri 839MP; Group G: Precizon Presbyopic; Group H: AcrySof IQ PanOptix; Group I: Tecnis Eyhanc

* There was one patient whose left eye was included in Group C and the right eye in Group D; ^†^ The first *P*-value is for the difference between 3 and 4 mm in the selected outcome among the groups, and the other due *P*-values are only referring to selected outcome at 3 or 4 mm, respectively

## Discussion

Recently, the impact of IOLs’ optic aberrations on retinal image quality is gaining increasing attention [3, 19, 20]. Significant correlations were found between visual acuity and ex vivo image quality metric for multifocal and monofocal IOLs, giving surgeons the opportunity to predict visual outcomes [3]. The far distance retinal image quality is of primary importance when considering neuroadaptation, as a decreased quality of image with blurred vision limits the neuroadaptation process. Neuroadaptation failure is mainly characterized by decreased quality of vision, sometimes with no correlation with the objective parameters of optical quality and no solid underlying reason such as posterior capsule opacification, dry eye, or retinal disease. The reduction in this far distance quality of vision is generally due to sensations of blurred vision, dysphotopsia or photic phenomena. Therefore, it is of primary concern how our brain reacts to a new input, such as what follows after implanting multifocal or EDoF lenses, and it is in good part related to the far distance retinal image quality, as a bad retinal image quality is inevitably a compromise and a limitation for the neuroadaptation process; however, other factors such as photic phenomena or the type of defocus curve are also considered to play a role in the tolerance to such atypical optics [6].

The most commonly used aberrometers are based on the Hartmann–Shack sensor: aberrometers based on this kind of working principle usually provide reproducible measurements in normal eyes but presents several limitations due to overlapping of the spots, especially in eyes with high levels of aberrations or after multifocal IOL implant: in the case of diffractive IOL, indeed, different replicas of the wavefront might be confusing [12]. The reconstructed wavefront and associated metrics are incorrect as all the information with regard to the add power of the diffractive lens are ignored [21]. The PWS-based aberrometer represents an important and novel tool that offers advantages for the purpose of these measurements [13]. To the best of our knowledge, the extensive evaluation of postoperative optical effect based on the study of the far distance PSF Strehl ratio obtained with a PWS-based aberrometer in pseudophakic eyes implanted with different IOLs has not been reported in peer-reviewed literature for all these studied lenses together and never explored before.

In this investigation, we evaluated the retinal image quality for the distance focus of a monofocal spherical IOL commonly used in clinical practice (AcrySof SA60AT), an EDoF (Miniwell), 3 multifocal refractive IOLs (LENTIS Mplus 15, LENTIS Mplus 30, Precizon Presbyopic), 2 multifocal diffractive IOLs (AT LISA Tri, AcrySof IQ Panoptix), an accommodative IOL (AkkoLens Lumina) and a new monofocal aspheric IOL (Tecnis Eyhance). The study obtained ocular aberrations using a new PWS-based aberrometer (Osiris) in all eyes at a pupillary diameter of 3.0 and 4.0 mm.

When considering the impact of IOLs on optical aberrations, one must consider how much light energy is intentionally and unintentionally directed off axis, especially when considering EDoF or multifocal—both diffractive and refractive—IOLs. In a monofocal IOL, the goal is to focus all light energy on axis in the plane of the retina. Therefore, in our control group implanted with a monofocal spherical IOL, we had a good retinal image quality (PSFw2 Strehl ratio 0.41 ± 0.11 and 0.28 ± 0.07 at 3.0 and 4.0 mm, respectively).

In the case of those aspheric multifocal lenses that increase depth of focus through spherical aberration, retinal optical quality in the far focus will inevitably be compromised, since the goal is to achieve a beneficial compromise between the gain in depth of focus and the loss in image quality [19, 22]. In fact, we found a drastic significant reduction in PSFw2 Strehl ratio values in the Miniwell group (0.28 ± 0.07 and 0.21 ± 0.06 at 3.0 and 4.0 mm, respectively; P < 0.0001): this data may partly explain the higher IOL exchange rate obtained in this group (31.6%).

In case of a rotationally asymmetric refractive multifocal lens, its vertical asymmetric optical geometry provides two distant foci for far and near vision by the presence of a calculated magnitude intraocular primary coma [23, 24]. Although some amounts of vertical coma have a positive effect on near visual acuity because of the enhanced depth of focus, high values of this aberration could limit the eye’s optical quality [25]. As expected, we found a trend toward a larger magnitude of HOA RMS in the far distance image in those eyes implanted with a 3.00 D posterior sector-shaped near-vision zone (Mplus MF30, group C: 0.30 ± 0.15 µm at 3.0 mm and 0.50 ± 0.12 µm at 4.0 mm). These findings suggest that the use of a larger add for the rotationally asymmetric IOL limits optical quality, with a relative effect on retinal image quality, as expressed by a significant lower value of PSFw2 Strehl ratio at both pupil diameters (0.23 ± 0.07 at 3.0 mm and 0.16 ± 0.05 at 4.0 mm; *P* < 0.0001).

Precizon Presbyopic is a continuous transitional focus IOL that obtains a smoother transition between distance and near vision by combining different sectors in the optical zone of the IOL [26, 27]. In our study, those eyes (group G) presented significant lower levels of retinal image quality (0.27 ± 0.07 and 0.17 ± 0.04 at 3.0and 4.00 mm, respectively; *P* < 0.0001) than the monofocal spherical group (SA60AT).

Another approach to correct near and far vision is (1) diffractive structure covering the entire anterior optical surface as in the AT LISA Tri 839 MP or (2) diffractive structure in the central 4.5 mm portion (15 diffractive zones) as in the AcrySof IQ PanOptix Trifocal, [28] to create three wavefronts of different curvatures emerging from the IOL: a regular flat wavefront for distance correction and two additional converging spherical wavefronts produced by the diffractive rings for near and intermediate vision [29]. These designs offer two of the highest mean postoperative value of retinal image quality in both groups, significantly higher than the control group for AT LISA Tri (Group F, *P* < 0.0001: 0.52 ± 0.14 and 0.31 ± 0.1 at 3.0 and 4.0 mm, respectively) but not significantly different than the monofocal control group for PanOptix (group H, *P* = 0.345: 0.4 ± 0.07 and 0.26 ± 0.04 at 3.0 and 4.0 mm, respectively).

The AkkoLens Lumina consists of two optical elements, which move one over the other in a plane perpendicular to the optical axis, aiming to produce a continuous variable-focus lens and change the dioptric power of the system while they change their position [16, 17]. We found a good level of PSFw2 Strehl ratio, even though significantly lower when compared to the monofocal spherical group (0.32 ± 0.11 and 0.23 ± 0.16 at 3.0 and 4.0 mm, respectively; *P* < 0.0001).

Finally, we also report herein for the first time the far distance retinal image quality in patients implanted with a Tecnis Eyhance. It is a new aspheric monofocal IOL that aims to enhance the image quality at intermediate distances without compromising distance vision, based on a continuous refractive optical surface design [30]. These patients had a relatively good retinal image quality (0.36 ± 0.11 and 0.21 ± 0.06 at 3.0 and 4.0 mm, respectively), but significantly lower than the monofocal spherical control group (*P* < 0.0001).

Finally, we compared those groups implanted with IOLs based on a similar basis for the far distance image. When comparing the values of PSFw2 between the diffractive IOLs, AT LISA Tri was found to provide a significantly better retinal image quality than PanOptix at both 3.0 mm (*P* < 0.0001) and 4.0 mm (*P* = 0.004), probably due to the aspheric design of the AT LISA Tri. Among the rotational asymmetric refractive IOLs, LENTIS Mplus MF15 was found to be significantly better than LENTIS Mplus MF30 at both 3.0 mm (*P* < 0.0001) and at 4.0 mm (*P* = 0.002). There were highly significant differences (*P* < 0.0001) among all groups regarding almost all postoperative ocular aberration components. This suggests that ocular aberration in pseudophakic patients were mainly caused by the optic of the implanted IOL. Consequently, studying ocular aberrations using a PWS-based aberrometer makes it possible to directly study the retinal image quality and thus guide the physician in IOL selection and in the future development of their patients’ optics.

The PWS-based aberrometer is a potentially good tool to evaluate the clinical quality of IOL performance when implanted in the human eye, evaluating the wavefront of the patient when looking at the infinity. Those lenses that are affected by the best optical outcome in the far distance focus with pyramidal aberrometry should also perform better in general.

A limitation of this study is that the post-hoc analysis could not be performed since we have several groups, which would make the results of such comparisons underpowered. Furthermore, one should consider that we cannot fully remove the influence of residual ametropia by removing the second-order aberrations, as there might be some interplay between the different Zernike modes. Finally, the Osiris – even if it has a higher resolution than previous aberrometers – is only able to simulate the main PSF of a diffractive lens, but not the others. Resolution considers the refractive quality of the diffraction pattern, but not the diffraction effect. In this study, measured wavefront error is the one of a patient looking at the infinity and its related PSF represent vision of a point for the far distance focus; in case of diffractive lenses, it is compressive all the aberrations eventually induced by the splitting system, but it does not comprehend the loss of contrast due to replicas. We focused our attention of the PSF of the main replica by evaluating if the splitting mechanism could affect the quality of the main focus. This might partly explain the high value of PSFw2 obtained with these two types of IOLs, that it was found to be comparable and/or higher than one of the monofocal IOLs.

## Conclusions

The diffractive multifocal AT LISA Tri showed the best far distance retinal image quality, significantly higher than the control group, followed by a not statistically significant near tie between the Alcon SA60AT monofocal spheric IOL and the Alcon Panoptix multifocal diffractive IOL. On the other hand, EDoF Miniwell, LENTIS Mplus MF30 and Precizon Presbyopic showed the significantly poorest retinal image qualities. When comparing IOLs whose optics had a similar design, AT LISA Tri had a significantly better far distance retinal image quality than PanOptix, and LENTIS Mplus MF15 had a significantly better retinal image quality than LENTIS Mplus MF30.

The explanation why lenses with diffractive optics, such as the diffractive IOLs included in this investigation, give higher values of far distance retinal image quality is out of the scope of this study. Learning how the different IOL optics influence the quality of retinal image by the study of induced aberrations with the novel PWS-based aberrometer, may be considered as a new clinical tool for IOL selection. It could influence IOL selection for the correction of pseudophakic presbyopia and guide surgeons in the evaluation and selection of the IOL to be implanted. Preoperative measurement of corneal HOA may be also important in sphericity inducing IOL to avoid explantation of these IOLs. Future prospective studies on this topic are warranted to correlate the findings of this report and to elucidate the relationship between far distance retinal image quality (assessed by the study of the PSF Strehl ratio), quality of vision perceived by the patient and the success of the neuroadaptation process that follow the implantation of lenses with multifocal or EDoF, in order to increase the optical quality of future lenses depending on their potential to create an adequate quality of retinal image and preventing the risk of neuroadaptation failure related to poor retinal image quality.

## Data Availability

All data analysed during this study are included in this published article and its supplementary information files.

## Abbreviations

CDVA: Corrected distance visual acuity
CNVA: Corrected near visual acuity
IOL: Intraocular lens
EDoF: Extended depth of focus
LOA: Low-order aberrations
HOA: High-order aberrations
PSF: Point spread function
PWS: Pyramidal wavefront sensor
UDVA: Uncorrected distance visual acuity
UNVA: Uncorrected near visual acuity
